# Reflex Sympathetic Dystrophy of the Right Hand following an Acute Traumatic Injury

**DOI:** 10.7759/cureus.5363

**Published:** 2019-08-11

**Authors:** Pooja Patel, Shweta Thadeshwar, Mausam Maru, Rupak Desai, John Fahey

**Affiliations:** 1 Rheumatology, Advocate Aurora Health, Brookfield, USA; 2 Miscallaneous, Nanavati Hospital, Mumbai, IND; 3 Public Health, Saint Louis University, Saint Louis, USA; 4 Cardiology, Atlanta Veterans Affairs Medical Center, Decatur, USA

**Keywords:** hand pain, swelling, pain, reflex sympathetic dystrophy, complex regional pain syndrome (crps), acute, trauma, tendon rupture, injury

## Abstract

Complex regional pain syndrome (CRPS), formerly known as reflex sympathetic dystrophy, is a chronic neuropathic pain disorder with significant autonomic features. Recently, it has been recognized that CRPS is not simply a sympathetically mediated peripheral pain condition but rather a disease of the central nervous system as well. Herein, we present a case of a patient who presented with complaints of severe pain following a traumatic event, severing his extensor tendon of his right fifth finger.

## Introduction

Complex regional pain syndrome (CRPS) is a disorder of a particular region of a body, usually involving the distal extremities, characterized by pain, swelling, limited range of motion, vasomotor instability, skin changes, and patchy bone demineralization [1]. It is frequently triggered following a fracture, soft tissue injury, or surgery. The pain is not restricted to a specific nerve territory or dermatome and usually has a distal predominance of abnormal sensory, motor, sudomotor, vasomotor, and/or trophic findings [2]. This syndrome shows variable progression over time.

## Case presentation

A 62-year-old, healthy male presented to the occupational health office with a chief complaint of new-onset, gradually worsening, localized, right-hand pain that began while he was at work. He is an employee at a steel factory, alternatively using a vibratory pressure hammer to clean and thin metals and a grinder for approximately nine hours a day, for the past seven years. The patient noticed increased discomforting pain in his right hand while he was at work, rating it 5 on a pain scale of 10. At the office, he reported difficulty flexing his fourth and fifth fingers in his right hand, unable to make a complete fist. The patient reported associated mild numbness of his right fourth and fifth fingers. His symptoms were aggravated with movement and relieved with rest. He denied taking any over the counter non-steroidal anti-inflammatory agents for pain relief. The patient denied any trauma or injury to his extremities. The patient denied knowledge of any other known contributing factors or events. The patient denied similar symptoms in his left hand. The patient reported that he was asymptomatic prior to this event. He denied any aches, pain, swelling or stiffness of his other joints. The patient reported unremarkable family and social history.

Upon physical examination, his vital signs were within normal limits. The patient was conscious, cooperative, well-developed, well-nourished, well-oriented in time, place and person. The patient had a full unrestricted range of motion of the left upper extremity. On evaluating his right hand and wrist, the patient had difficulty fully flexing his right fourth and fifth fingers, unable to make a complete fist. The patient had an unremarkable extension of his fingers. Capillary refill was less than two seconds bilaterally. The patient had unremarkable Tinel’s and Phalen’s signs, bilaterally. 

The patient was diagnosed with work-related acute traumatic injury to his right hand. He was suspected to have a possible flexor tendon injury and hence was referred to occupational therapy and a hand-orthopedic surgeon for further evaluation. In the interim, he was recommended taking non-steroidal anti-inflammatory agent, with food as needed for pain. The patient was educated and taught a gentle range of motion exercises. He was provided a work restriction of avoiding any lifting, pushing or pulling greater than 10 pounds or using any vibrating tools. 

During his occupational therapy appointment, his symptoms had improved mildly in intensity; averaged his pain to be 2-5 on a pain scale of 10. The patient expressed his desired therapy goal would be to regain his ability to bend his finger like his other hand. After a thorough examination, therapy goal was established and continue following-up for therapy one time per week for four weeks (Table 1).

**Table 1:** Occupational therapy goals

Goals to be obtained at the end of occupational plan of care
Patient will be independent with his activities
Decreased involved hand pain to 0 out of 10
Achieve active fingertip to palm composite flexion for resumption of small object grasp and hold and ½ inch diameter tool use (toothbrush, eating utensils)
Achieve sufficient pinch/ dexterity
Patient be able to lift 50 pounds from floor to waist with proper body mechanics to assist with lifting at work
Patient be able to lift 10 pounds with proper body mechanics to assist with lifting overhead activities at work

**Figure 1:**
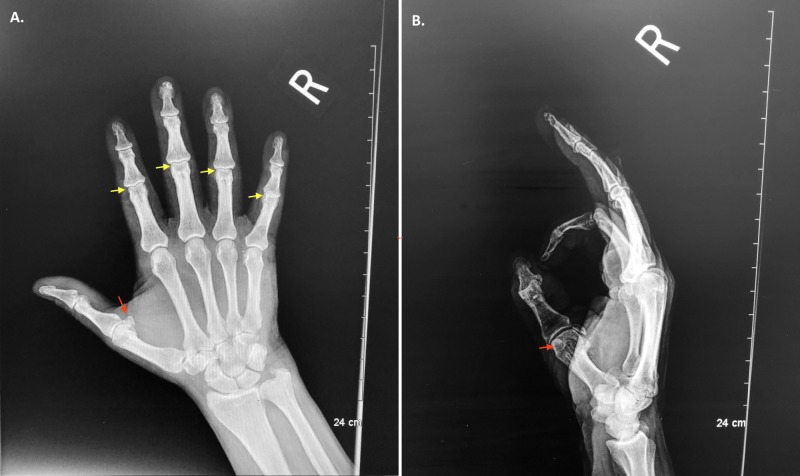
X-ray image of the right hand (two views) Findings: X-rays of the right hand demonstrate a mild hypertrophic change of the head of the first metacarpal (red arrow). Mild narrowing was evident at the proximal interphalangeal joints, with mild associated hypertrophic changes (yellow arrow). Impression: Mild early osteoarthritic changes; no evidence of fracture or dislocation

On evaluation by the hand-orthopedic surgeon, the patient was noted to have full flexion of his metacarpophalangeal joints in his right hand, including his right fifth finger, but there was no active flexion at his right fifth proximal or distal interphalangeal joints. He had full passive range of motion of his right fifth finger, but he could not hold his finger bend at all at his distal and proximal interphalangeal joints. Comparing his left hand, he was able to make a full fist on his left hand with full flexion of all his fingers. X-ray image of his hand was obtained (Figure 1).

**Figure 2:**
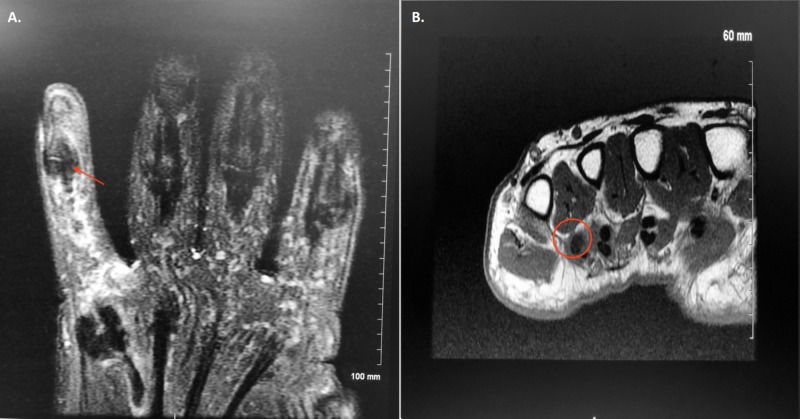
MRI scan of the right hand without contrast Impression: Complete tear involving the flexor digitorum profundus and superficialis tendons to the fifth digit located at the level of the mid-fifth metacarpal (red arrow). The proximal tendon stumps are not well visualized in the field-of-view. If further evaluation more proximally is clinically indicated, MRI of the wrist with the inclusion of the proximal metacarpals could be considered. There is some mild strand-like elevated transverse plane (T2) signal near the distal remaining tendons which may be due to edema or bruising (red circle). There is some strand-like elevated T2 signal in the subcutaneous tissues around the fifth digit which may be due to edema or bruising.

The surgeon suspected rupture of his flexor digitorum profundus tendon to his right fifth finger. His palmaris longus tendon was intact, which was helpful to know for a possible reconstruction surgery in the future. To further investigate the cause of his symptoms, a magnetic resonance imaging (MRI) scan of his right hand was ordered (Figure 2).

The rheumatologist reviewed the patient’s history in detail with him. He denied any autoimmune conditions or gout in his family. On physical examination, the patient had 1+ swelling of right hand with tenderness on palpation. He had mildly increased warmth of his right hand without erythema. He could make a fist with his right hand only by 30 degrees. 

**Figure 3:**
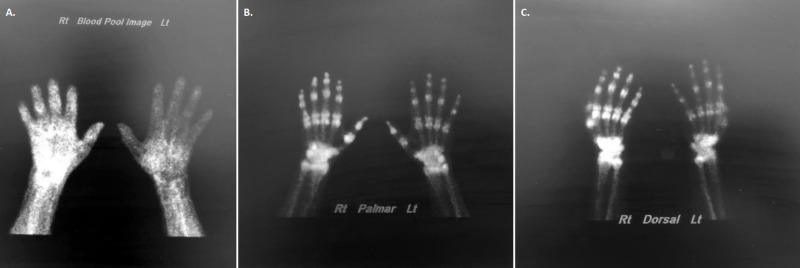
NMBS of the right hand Findings: The flow study indicates subtle increased flow diffusely throughout the visualized distal right forearm, wrist and hand. There is normal flow through the distal left forearm wrist and hand (Image A). In the blood pool images corresponding to the flow images, there is diffusely increased radiotracer throughout the soft tissues of the distal right forearm, wrist, and hand. There is normal uptake of the radiotracer in the left distal forearm wrist and hand (Image B). The delayed scan showed asymmetric diffusely increased periarticular uptake of joints of the wrist, carpometacarpal joints, metacarpophalangeal joint, and interphalangeal joints of the right hand. There is normal uptake of the visualized portions left upper extremity (Image C). Impression: Three-phase radiotracer uptake of the right distal forearm, wrist, and hand with diffuse periarticular delayed uptake of the wrist and hand. Findings are compatible with reflex sympathetic dystrophy. NMBS, nuclear medicine bone scan

The rheumatologist concluded that the patient presented with active synovitis of his right hand. The differential diagnosis for his symptoms included inflammatory arthropathy, autoimmune arthropathy, Lyme’s disease, reflex sympathetic dystrophy, or gout. Laboratory tests were ordered, along with a nuclear medicine bone scan (NMBS) to evaluate the bone activity in his right hand (Table 2, Figure 3).

**Table 2:** Laboratory test results

Component	Latest Reference, Range and Unit	Result
White Blood Count	4.2–11 thousand per microliter	5.2
Red Blood Cell	4.50–5.90 million per microliter	4.86
Hemoglobin	13.0–17.0 grams per deciliter	15.4
Hematocrit	39.0–51.0 percent	44.7
Mean Corpuscular Volume	78.0–100.0 fluid ounce	92.0
Mean Corpuscular Hemoglobin	26.0–34.0 picogram	31.7
Mean Corpuscular Hemoglobin Concentration	32.0–36.5 grams per deciliter	34.5
Red Blood Cell Distribution Width	11.0–15.0 percent	13.1
Platelet	140 – 450 thousand per microliter	143
Nucleated Red Blood Cell	0 per 100 white blood cells	0
Erythrocyte Sedimentation Rate	0-20 millimeter per hour	6
Albumin	3.6–5.1 grams per deciliter	4.0
Total Bilirubin	0.2–1.0 milligram per deciliter	0.7
Direct Bilirubin	0.0–0.2 milligram per deciliter	0.2
Alkaline Phosphatase	45–117 units per liter	122 (H)
Aspartate Aminotransferase	<79 units per liter	169 (H)
Alanine Aminotransferase	<38 units per liter	87 (H)
Total Protein	6.4–8.2 milligram per deciliter	7.5
Creatinine	0.64–1. 17 milligram per deciliter	0.78
Estimated Glomerular Filtration Rate, African American	>59.9 milliliter per minute per 1.73 square meter	>90
Estimated Glomerular Filtration Rate, non-African American	>59.9 milliliter per minute per 1.73 square meter	>90
Cyclic Citrulline Peptide Antibody	<20 units	14
Uric Acid	3.5–7.2 milligram per deciliter	5.3
Antinuclear Antibody	Negative	Negative
C-Reactive Protein	<1.0 milligram per deciliter	<0.3
Borrelia Burgdorferi Antibody Screen	Negative	Negative
Rheumatoid Factor	<15 units per milliliter	<10

Following the NMBS results, the patient was referred to pain management for further treatment, in anticipation of a possible stellate ganglion nerve block. The patient showed slow symptomatic improvement while he continued following-up with occupational therapy, and was recommended to avoid repetitive vibratory or jarring activities. 

The patient deferred undergoing stellate ganglion nerve block procedure for pain relief. He successfully completed his occupational therapy sessions with mild symptomatic improvement. He was further referred to physical therapy in anticipation of desensitization therapy and started on Gabapentin 300 milligram capsules three times daily to help manage his pain.

## Discussion

Older alternative names for CRPS in the literature include reflex sympathetic dystrophy (RSD), algodystrophy, causalgia, Sudeck atrophy, transient osteoporosis, and acute atrophy of bone. Upper extremity involvement following the stroke or myocardial infarction was sometimes referred to as the "shoulder-hand syndrome." In the modern era, these disorders are grouped under the single heading of CRPS. 

There are two families of classification of CRPS; based on the presence or absence of nerve injury and based on skin temperature during the onset of symptoms (Table 3) [2-4].

**Table 3:** Classification of CRPS CRPS, complex regional pain syndrome

Based on nerve injury:	
Type 1	- Also known as reflex sympathetic dystrophy; corresponds to patients with CRPS without evidence of peripheral nerve injury and represents approximately 90 percent of clinical presentations.
Type 2	- Formerly termed "causalgia"; refers to cases in which peripheral nerve injury is present.
Based on skin temperature:	
Warm	- Increased skin temperature at the onset of symptoms, suggestive of inflammatory type CRPS
Cold	- Decreased skin temperature at the onset of symptoms

Evidence for inflammation in the presence of an increased level of cytokines interleukin (IL)-6, IL-1 beta (1β), and tumor necrosis factor-alpha (TNF-α) from the venous blood samples of the involved versus uninvolved extremity in the acute phase suggesting a local inflammatory process [5]. Another possible explanation for pain and allodynia in CRPS involves central sensitization, whereby increased activity in nociceptive afferents due to peripheral noxious stimuli, tissue damage, or nerve injury leads to increased synaptic transmission at somatosensory neurons in the dorsal horn of the spinal cord [6]. 

The main clinical symptoms of CRPS are pain, sensory changes, motor impairments, autonomic symptoms, and trophic changes in the affected limb [7]. The onset of symptoms occurs generally within four to six weeks of the inciting event. The most common inciting events leading to CRPS are fractures, crush injuries, sprains, and surgery. 

There is no "gold-standard" test or method for confirming the diagnosis. However, some investigators assert that certain investigations are useful for the diagnosis of CRPS, particularly three-phase bone scintigraphy showing increased radiotracer uptake in joints distant from the trauma site. Other tests with some possible utility include side-by-side radiographs (e.g., both hands imaged on the same radiograph) showing spotty bone decalcification, and long-term or repetitive skin temperature measurements showing >1 °C difference for the affected versus unaffected side [7]. There is no clear role for MRI or computed tomography (CT) scanning in the evaluation of suspected CRPS, nor is there any role for the response to sympatholysis to confirm the diagnosis of CRPS. The Budapest consensus criteria have been described for the clinical diagnosis of CRPS (Table 4) [8].

**Table 4:** Budapest consensus criteria for the clinical diagnosis of complex regional pain syndrome *A sign is counted only if it is observed at the time of diagnosis

Budapest consensus criteria for clinical diagnosis of complex regional pain syndrome
1. Continuing pain, which is disproportionate to any inciting event
2. The patient must report at least one symptom in three of the following four categories: Sensory: Reports of hyperesthesia and/or allodynia vasomotor: Reports of temperature asymmetry and/or skin color changes and/or skin color asymmetry Sudomotor/edema: Reports of edema and/or sweating changes and/or sweating asymmetry Motor/trophic: Reports of decreased range of motion and/or motor dysfunction (weakness, tremor, dystonia) and/or trophic changes (hair, nail, skin)
3. The patient must display at least one sign* at the time of evaluation in two of the four following categories: Sensory: Evidence of hyperalgesia (to pinprick) and/or allodynia (to light touch and/or temperature sensation and/or deep somatic pressure and/or joint movement) Vasomotor: Evidence of temperature asymmetry (>1°Celsius) and/or skin color changes and/or asymmetry Sudomotor/edema: Evidence of edema and/or sweating changes and/or sweating asymmetry Motor/trophic: Evidence of decreased range of motion and/or motor dysfunction (weakness, tremor, dystonia) and/or trophic changes (hair, nail, skin)
4. There is no other diagnosis that better explains the signs and symptoms
* A sign is counted only if it is observed at the time of diagnosis

CRPS can mimic various other medical conditions, delaying diagnosis and hence, appropriate management. The differential diagnoses are summarized in Table 5 [9].

**Table 5:** Differential diagnosis of complex regional pain syndrome N/A= Not Applicable

Diagnosis	Clinical presentation	Investigations	Key points for chronic regional pain syndrome
Infection of skin, muscle, joint, or bone	Redness (erythema), swelling (edema), warmth, and pain	Elevated erythrocyte sedimentation rate, or C-reactive protein, and elevated white blood cell count in the peripheral blood	The investigations are all normal for chronic regional pain syndrome
Compartment syndrome	Early symptoms include progressive pain out of proportion to the injury; signs include tense swollen compartments and pain with passive stretching of muscles within the affected compartment	Surgical emergency	N/A
Gout	Acute onset pain, swelling and erythema of the joint with/without increased warmth	Elevated erythrocyte sedimentation rate, C-reactive protein, uric acid level, and synovial fluid examination showing evidence of uric acid crystals or monosodium crystals	Chronic regional pain syndrome would not elevate uric acid level
Deep vein thrombosis	Swelling, redness, and pain of the extremity involved	Doppler ultrasound vascular testing	Can differential it from deep venous thrombosis based on history and physical examination, and doppler ultrasound vascular study
Peripheral neuropathy	Hypersensitivity and dystrophic changes of the extremities	Electromyographic study	N/A
Rheumatoid arthritis	Active inflammation or synovitis of multiple joints	Rheumatoid factor, cyclic citrulline peptide, erythrocyte sedimentation rate, C-reactive protein	Usually involves one region of the body
Raynaud’s phenomenon	Sharply demarcated color changes of the skin (to red, white, blue, or combination of colors) of the digits.	The diagnosis of Raynaud phenomenon is made if the fingers are unusually sensitive to cold and change color when exposed to cold temperatures.	Thorough history and physical examination
Conversion disorder	An involuntary condition in which neurologic symptoms are present in the absence of neurologic disease but are not feigned	N/A	Thorough history and physical examination
Factitious disorder	An intentional production of physical or psychological symptoms or findings to assume the "sick role."	Thorough history and physical examination	N/A

Management of CRPS should include a multidisciplinary approach. The most vital intervention in the management of CRPS is inclusion of patient education and early introduction of physical and occupational therapy. The therapeutic management goal is to allow active participation in the rehabilitation regimen and restore movement and strength of the affected limb. Clinical based therapeutic interventions have been summarized in (Table 6) [10].

**Table 6:** Management of complex regional pain syndrome N/A = Not Applicable *Sign is counted only if it is observed at the time of diagnosis

Treatment	Recommendation	Note
Pharmacological approach		
Non-steroidal anti-inflammatory drugs	- Ibuprofen 400-800 milligrams, three times a day - Naproxen 500 milligrams, twice daily - Ketorolac 60 milligrams intravenously	Often used as initial treatment in the management of chronic regional pain syndrome
Anticonvulsants	- Gabapentin up to 1800 milligrams daily - Pregabalin - Carbamazepine	Beneficial for neuropathic pain
Antidepressants - serotonin-norepinephrine reuptake inhibitor - tricyclic antidepressants	- Amitriptyline - Nortriptyline	
Opioids	N/A	Insufficient clinical evidence showing beneficial effects of morphine infusion
Bisphosphonates	- Alendronate (oral) 40 milligrams a day for 8 weeks - Clodronate (intravenous) - Pamidronate (intravenous)	- Bisphosphonates have beneficial effect on the signs* of inflammation - Should be considered in patients with increased/elevated bone metabolism
Muscle relaxants	- baclofen (oral) - diazepam - clonazepam - botulin toxin	Should be considered in patients with symptoms of dystonia, myoclonus or muscle spasms
Corticosteroids	Oral glucocorticoids, example. prednisone	If high dose of glucocorticoid is initiated, the patient should be gradually tapered down on the dose of glucocorticoid to avoid acute adrenal crisis
Interventional procedures:		
Regional sympathetic nerve block	- Temporary sympathetic nerve block can be performed by administering local anesthesia into the region of the sympathetic ganglia - Intravenous regional sympathetic blocker infusion in combination of a local anesthetic	- Helps improve circulation in patients with cold chronic regional pain syndrome - Stellate ganglion block can be performed
Spinal cord stimulation	N/A	Should be considered when traditional therapeutic modalities fail
Sympathectomy	N/A	Side effects include hyperhidrosis and neuropathic complications

Vitamin C has been recommended to reduce the prevalence of CRPS in patients with wrist fracture [11]. The prognosis of chronic regional pain syndrome is highly variable, depending on the patient’s symptomatic complaints. The incidence of recurrence is low and more than 50% of the recurrences of chronic regional pain syndrome are spontaneous in origin, especially in younger patients [12].

## Conclusions

CRPS is a multifactorial complex disorder of the extremities, with regional pain disproportionate to the mechanism or extent of the injury. It is a chronic neuropathic pain (burning, hyperalgesia, and allodynia) associated with local edema and autonomic changes inclusive of altered sweating, skin temperature or skin color. The diagnosis is based on history and physical examination, and the multidisciplinary approach to symptomatic patient management with CRPS should be initiated.
